# The complete mitochondrial DNA genome of a cone snail, *Conus betulinus* (Neogastropoda: Conidae), from the South China sea

**DOI:** 10.1080/23802359.2021.1930212

**Published:** 2021-05-19

**Authors:** Yanling Liao, Jinxing Fu, Bingmiao Gao, Tianle Tang

**Affiliations:** Key Laboratory of Tropical Translational Medicine of the Ministry of Education, Hainan Medical University, Haikou, China

**Keywords:** Cone snail, *Conus betulinus*, mitochondrial genome, phylogenetic analyses

## Abstract

The complete mitochondrial genome of the tubular cone snail *Conus betulinus* is presented in this study. The *C. betulinus* mitochondrial genome was 16,240 bp with 13 protein-coding genes (PCGs), 22 transfer RNA (tRNA) genes, 2 ribosomal RNA (rRNA) genes, and a non-coding AT-rich region (D-loop). The overall base composition was estimated to be 25.67% for A, 38.26% for T, 21.38% for G, and 14.69% for C, with a high A + T content of 63.93%. Phylogenetic analyses based on 13 PCGs showed the close relationship of vermivorous *C. betulinus* with the common ancestor of molluscivorous *Conus textile* and *Conus gloriamaris*, providing a basis for further studies on the phylogenetics of cone snails according to their dietary type.

Members of the genus *Conus* (Neogastropoda: Conidae), poisonous carnivores that live in tropical ocean waters around the world, produce complex conotoxins for hunting and defense. More than 700 extant species and 57 subgenera are recognized in this classification (Puillandre et al. 2014; Gao, Peng, Chen, et al. 2018). As the largest genus among marine invertebrates, cone snails can be divided into the following three groups based on their diet: piscivorous, molluscivorous, and vermivorous (Gao et al. 2017). Vermivorous species account for 70% of the genus, and some studies have been conducted on the dominant vermivorous species that seem to be non-threatening (Himaya et al. 2015; Peng et al. 2016). In recent years, researchers have attempted to elucidate the evolutionary relationship between venom and feeding from population genetics, evolutionary biology, and phylogenetics (Aman et al. 2015; Gao, Peng, Chen, et al. 2018).

We assembled and characterized the complete mitochondrial genome sequence of vermivorous *Conus betulinus* Linnaeus 1758 (Neogastropoda: Conidae) to provide information for the identification of *Conus* and support further phylogenetic studies of Neogastropoda. Live *C. betulinus* was collected from the offshore areas of Sanya City, Hainan Province (18°09N, 108°56E). The sample was deposited at the Key Laboratory of Tropical Translational Medicine of the Ministry of Education, Hainan Medical University, Haikou, Hainan, China. The specimen accession number was CHMU0119. Total genomic DNA was extracted using the Column mtDNAout kit (Tianda, Beijing, China), and the purified genomic DNA was characterized using the Nanodrop 2000 spectrometer (Thermo Fisher Scientific, Wilmington, DE, USA). The NEBNext DNA Library Prep Kit (New England Biolabs, Ipswich, MA, USA) was used to establish a paired-end library. Quantification and sizing of the library were performed on a Bioanalyzer 2100 High Sensitivity DNA chip (Agilent, Palo Alto, CA, USA). The library was normalized to 2 nM and sequenced on the Illumina HiSeq2000 platform (Illumina, San Diego, CA, USA). The mitochondrial genome was assembled using SPAdes v.3.5.0 (Bankevich et al. 2012) with k-mer size 79, the average sequencing depth of mitochondrial genome is 85× and coverage 100% of the genome. Gene annotation was performed using the online programs Dual Organellar GenoMe Annotator (DOGMA) (Wyman et al. 2004), ORF Finder (Cheng et al. 2013), tRNAscan-SE (Schattner et al. 2005), and ARWEN with the annotated results of ARAGORN (Abe et al. 2011). All protein-coding genes (PCGs), transfer RNAs (tRNAs), ribosomal RNAs (rRNAs), and a non-coding AT-rich region (D-loop) were confirmed using Basic Local Alignment Search Tool (BLAST) (Altschul et al. 1990), tRNAscan-SE, and ARWEN (Griffiths-Jones et al. 2003; Abe et al. 2011).

The mitochondrial genome was a circular molecule of 16,240 bp in size with 13 PCGs, 22 tRNA genes, 2 rRNA genes, and a D-loop (GenBank accession number: MG924728). Eight tRNA genes were encoded on the light strand (*tRNA-Met*, *tRNA-Tyr*, *tRNA-Cys*, *tRNA-Trp*, *tRNA-Gln*, *tRNA-Gly*, *tRNA-Glu*, and *tRNA-Thr*), and the other 30 genes were located on the heavy strand. The overall base composition was estimated to be 25.67% for A, 38.26% for T, 21.38% for G, and 14.69% for C, with a high A + T content of 63.93%. The D-loop region between tRNA-Phe and *cox3* in *C. betulinus* was the longest (793 bp) among the regions of the *Conus* species, which had a higher A + T content (69.86%). The genes order of *Oxymeris dimidiate* (NC_013239), *Fusiturris similis* (NC_013242), and *C. betulinus* are coincident, except for tRNA-Val and tRNA-Ser, and they were major rearrangement were found in Neogastropoda (Duda 2001; Xing and Lee 2005; Bandyopadhyay et al. 2008).

To investigate the phylogenetic position of *C. betulinus* within *Conus*, the 13 PCGs of mitogenomes from 9 *Conus* species and 3 allochthonous species were downloaded from GenBank, and phylogenetic trees were constructed using the maximum-likelihood (ML) method with RAxML (version 8.1.5) (Stamatakis 2006). Vermivorous cone snails were clustered at the root of the tree, implying the cone snail ancestor may be vermivorous (Gao, Peng, Zhu, et al. 2018). However, the vermivorous clade is not monophyletic but paraphyletic to the other two dietary types. It can be seen that vermivorous *C. betulinus* was closely related to the common ancestor of molluscivorous *Conus textile* and *Conus gloriamaris* (Figure 1). The complete mitochondrial genome of the tubular cone snail *C. betulinus* will provide essential data for future research on the phylogenetic and evolutionary relationship in genus of *Conus*.

**Figure 1.**
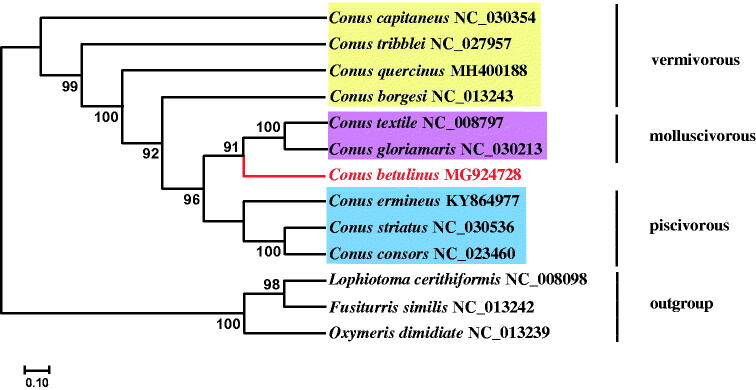
A maximum-likelihood (ML) phylogenetic tree based on 13 protein-coding genes from 13 conid species constructed using RAxML. All branch nodes are indicated with 1000 bootstrap replicates.

## Data Availability

The data that support the findings of this study are available in the NCBI database at https://www.ncbi.nlm.nih.gov, reference number MG924728.
